# “*They fell through the cracks:*” caregiver perspectives on the difficulties of COVID-19 implementation transitions for children and youth with special healthcare needs (CYSHCN)

**DOI:** 10.3389/fped.2025.1440680

**Published:** 2025-06-02

**Authors:** D. Grace Smith, Mary Kathryn Cancilliere, Tara M. Hayes, Anashua Rani Elwy, Indra Neil Sarkar

**Affiliations:** ^1^ Division of Biology and Medicine, Brown University, Providence, RI, United States; ^2^ Department of Psychiatry and Human Behavior, Brown University, Providence, RI, United States; ^3^ Department of Child and Adolescent Psychiatry, Rhode Island Hospital, Providence, RI, United States; ^4^ Rhode Island Parent Information Network, Warwick, RI, United States; ^5^ Rhode Island Quality Institute, Providence, RI, United States

**Keywords:** children with special healthcare needs, CYSHCN, COVID-19, pandemic, vaccination, implementation barriers.

## Abstract

**Introduction:**

The COVID-19 virus and its relevant prevention protocol had significant impacts on children and youth with special healthcare needs (CYSHCN), including those with physical, intellectual, and developmental disabilities. Previous studies have examined the first year of the pandemic, identifying the implications of social distancing, remote services/education, and masking and testing protocol on the mental, physical, and developmental well-being of this population.

**Objectives:**

We focus on moments of transition, when vaccines were disseminated and mandates/resources removed. By identifying how protocol and interventions in these moments included, neglected, or negatively impacted CYSHCN, we can inform more inclusive, safe, and equitable decision-making in future health crises.

**Methods:**

We report the transition-moment-related reflections of fourteen focus groups conducted among caregivers (including parents) of CYSHCN between March and December of 2022 (*n* = 77). Focus groups were conducted in close partnership with a local non-profit organization, and facilitation protocol were co-developed by community leaders in the CYSHCN area. Focus groups were recorded, transcribed, and analyzed using directed content analysis and thematic analysis, guided by implementation science theories on interventions’ relative dis/advantages and perceived adaptability.

**Results:**

Caregivers provided transition-moment reflections of how the timing, support, and in/flexibility of pandemic intervention implementation and de-implementation negatively impacted their CYSHCN. We generated three themes to describe these views: (1) “*Our kiddos didn’t have a plan when this happened*:” Lack of transition support into isolation meant loss of necessary structures and services; (2) “*He couldn’t comprehend*:” Transition communication, particularly surrounding mandates and protocol, was not handled well for CYSHCN; and (3) “*Listen, we’re still in the middle of pandemic*:” Transition timing neglected consideration of CYSHCN.

**Discussion:**

CYSHCN needs were neglected in the pandemic’s transition moments, creating significant implications for their mental/emotional, physical, and cognitive/developmental well-being. Reflecting these findings, and particularly facets that extend established literature, we urge inclusive research and policy models, empowering members of the CYSHCN community as leaders in knowledge and protocol production, particularly when considering the adaptability and relative advantage of interventions. Such models are crucial in developing messaging around pandemic policies, creating infrastructural support for flexibility, and adding supports and delays when de-implementing mandates.

## 1 Introduction

The coronavirus pandemic (COVID-19) and its related intervention procedures (i.e., social distancing, masking, testing, remote learning, and vaccination) dramatically shaped the physical, mental, and social well-being of individuals across the globe for over two years (1–3). The impact of COVID-19 on children and youth has been well documented: while researchers have established that youth were overall less susceptible to serious physical harm upon contracting the virus (4), they have also noted that, because of developmental and related vulnerabilities, youth experienced difficulties in mental health (5, 6) and quality of life (2) as a result of the protocol implemented during the pandemic. Within these considerations, COVID-19 and pandemic protocol had particularly significant impacts on children and youth with special healthcare needs (hereafter, CYSHCN), defined as those who have been diagnosed with physical, developmental, or intellectual disabilities (7). These needs often create vulnerabilities to serious illness, inability to navigate daily life without support, and barriers to communication and comprehension (7).

Reflecting this, many studies have examined the implications of implementing virus prevention protocols among CYSHCN and related populations during the COVID-19 pandemic (1, 3, 8, 9). They have established various facets within the mechanisms of interventions requiring adaptation or exception because they were inaccessible, created harm, or debilitated the emotional/social wellbeing of community members (1, 3, 8, 9). For example, studies have shown that various diagnoses create barriers to people’s abilities to wear masks on account of breathing difficulties, seizure risk, and skin or related sensitivities (1, 8). Likewise, vaccines were inaccessible to or caused harm among some CYSHCN because they created risks of events like seizures, or because those with communicative difficulties were not able to express their experience of side effects necessitating immediate care (1, 7). Many adaptations implemented to allow for social distancing, such as remote learning and remote healthcare options, were also ineffective for CYSHCN because of difficulties maintaining focus, communicating nonverbally, and technological barriers, and, as a result, many of these interventions caused social, emotional, and communicational regression (1, 3). In the Rhode Island context, there is a history of limited additional supports that are made available for CYSHCNs by healthcare policies and federal/state organizations; these supports include expanded telehealth services, federal insurance waivers, and care coordination initiatives (10–12). Previous studies have found that the COVID-19 pandemic exacerbated existing healthcare disparities for CYSHCN, leading to increased unmet mental health needs, disrupted access to routine and specialty care, and financial strain on families (13, 14). Supports for families and CYSHCNs were often provided by a combination of state/federal sources and local non-profit organizations, causing local access to support in Rhode Island to be very variable, often determined by regional resources, socioeconomic status, rural/urban location, and fit of child’s needs with the available resources (11–13).

Because of the rapid spread of both COVID-19 and knowledge about it, interventions were disseminated and mandates made rapidly, often without flexibility or adequate accommodations. Multiple studies identified the impacts of implementation, de-implementation, and re-implementation of interventions in terms of resulting contagion rates and population medical implications (15–17), and policy and opinion publications argued prospectively against the early de-implementation of mandates (18–20). Most relatedly, Croft and Fraser, in a scoping review of literature (21) discussed the global experiences, dating back to the start of the pandemic (2020), of adults and youth with disabilities through the pandemic. These authors highlighted the first nine months of pandemic responses on the lives of people with disabilities (of all ages, globally) including the barriers and facilitators and similarly recognized the importance of identifying realities created by COVID-19 response measures, beyond the virus itself.

Here, we seek to expand the extant research beyond the variety of specific impacts, barriers, and implications within the mechanisms of pandemic interventions by exploring the effects of decisions made and methods employed at the level of implementation. We explored a high-needs community’s experiences of pandemic protocols, focusing on moments of transition across pandemic interventions (masks, vaccines, and distancing). Toward this end, we examined the lived experiences of caregivers (primarily parents and grandparents) of CYSHCN, inviting retrospective reflections in order to more fully understand the significant transition moments across the scope of the pandemic. By identifying how changes in pandemic-period protocols included, neglected, or negatively impacted CYSHCN, we can inform more inclusive, safe, and equitable decision-making and mandate-implementation in future health crises.

## 2 Methods

### 2.1 Overview

The National Institute of Health's RADx-UP initiative called for studies on COVID-19 testing, vaccination, and outcomes among underserved populations (22). This study was part of a statewide tracking, exploratory, and implementation effort aiming to (1) leverage health information infrastructure to study COVID-19 test/vaccine access and uptake in Rhode Island, (2) identify barriers to uptake among marginalized and high-needs communities (23), and (3) identify opportunities to address these barriers via a community-based approach. This manuscript reports on the second of these aims. We focus on the population of CYSHCN by gathering the perspectives of their primary caregivers, which included parents, in a series of focus groups. Brown University’s Institutional Review Board approved all research procedures.

### 2.2 Procedure

Using a community-centered model, this study was implemented in close partnership with a large local, non-profit organization that serves families of CYSHCN by providing education and programming, as well as resources, advocacy, and assistance with accessing medical, financial, educational, and other necessary supports. Staff members of the organization, trusted among the community, supported the study by recruiting participants using IRB approved strategies (through social media, flyers, and peer-to-peer engagement), guiding the development of the focus groups guide (see Table 1), and joining focus groups. Questions and prompts that guided the conversation were designed in collaboration with the staff members to elicit unbiased opinions from participants, based on the experience of the staff members with the community. Focus groups were facilitated by members of the research team trained in facilitation. Participants were recruited via convenience sampling, with recruitment methods including flyers distributed both at community events and online through social media. Caregivers were eligible for participation if they were parent or legal guardian of at least one CYSHCN with a connection to the organization (e.g., receiving services, resources), were residents of Rhode Island, and were legal adults (18+). All participants passed a manual vetting procedure conducted by non-profit staff to ensure match to inclusion criteria and eliminate repeated participation and “bot” registration. Participants provided informed consent through Zoom registration and verbal agreement, andwere compensated for their time with gift cards.

**Table 1:** Focus group key questions.

Item	Focus group question
1	What is your current opinion of the pandemic?^a^
	*Probes: What is the current opinion of others in your home? Friends and neighbors?*
2	What resources do you use to learn about the pandemic? How do you access those?
3	What are your thoughts on the mask requirements?
	*Probes: When do you and your children choose to mask/not mask? When do you think others should/not? Has your opinion changed since the vaccine?*
4	What are your thoughts on COVID-19 tests?
	*Probes: How would you access a test if you needed to? How would your behavior change in response to a positive/negative result?*
5	What are your thoughts on the vaccine?
	*Probes: If your children have received the vaccine, what was the experience like? If you haven’t, why? What can you share about your family’s, friend’s, neighbor’s experiences?*
6	Have your children experienced any social or mental health-related impacts throughout the pandemic?
	*Probes: Early on in the pandemic? More recently?*
7	How did the pandemic affect the number and kind of resources/services your child needed and accessed? What barriers did you face, if any?

Guide followed to structure the focus group conversations.

^a^ In discussing their current opinions, participants often reflected on how those views had changed over the course of the pandemic.

Focus groups took place virtually between March and December of 2022 and lasted between 60 and 90 min long. The goal of focus groups, concomitant with the RADx-UP overall project goals for the U.S., was to gather the retrospective perspectives of caregivers of CYSHCN on their experiences receiving mandated interventions, e.g., tests and vaccines, for COVID-19 from the start and throughout the pandemic.

### 2.3 Data analysis

Focus groups were recorded through the video conferencing platform Zoom, and the audio was transcribed and anonymized by a third-party service. The research team conducted analysis in two phases, employing methods similar to those published by this team elsewhere (23). First, the team conducted a directed content analysis (24) guided by the original Consolidated Framework for Implementation Research [CFIR; (25)]. The CFIR’s five domains are: (1) intervention characteristics, (2) outer context, (3) inner context, (4) characteristics of individuals targeted in implementation, and (5) implementation process (25). Trends in the data prompted the development of additional (inductive) codes (24). Analysts individually coded each transcript using this framework and, to establish consistency and rigor, cross-coded the transcripts in pairs. The first author led the consensus process by which final coding decisions were made and entered coded data into the qualitative data management software NVivo (23, 36).

The second phase of analysis was thematic (26). Because of the focus on aspects of pandemic interventions and implementation that created specific positive or negative experiences for CYSHCN, the analyst examined only data coded in two categories within the CFIR domain Intervention Characteristics. The first of these, Relative Advantage, is defined in this context as the perceived advantages in efficiency, efficacy, visibility of benefits, or any other factors of participating in an intervention over alternatives (25, 27, 28). The second, Adaptability, describes the ways in which an intervention can, cannot, or should be changed or refined according to community needs (25, 26, 27). In this paper, we examined comments about the adaptability of both the intervention itself and the implementation process—e.g., vaccine dissemination and enforcement (21, 28,29,34,35). Following the process of thematic analysis, the analyst read the data labelled with these two codes and developed themes that described key patterns. Iteratively rereading the data and drafting summary trends, the analyst reviewed, defined, and named the themes, and selected illustrative participant quotations (26).

## 3 Results

Between March and December of 2022, the research team conducted 13 focus groups. They varied in size from three to nine participants. In total, 77 caregivers of CYSHCN participated, 73 identifying as female and four as male. All participants were parent or legal guardian to at least one CYSHCN, and frequently mentioned diagnoses represented a wide range of intellectual, physical, and emotional diagnoses (e.g., autism, attention deficits, cerebral palsy and motor disorders, anxiety and obsessive compulsive disorder, etc.) No participants were excluded during or after their focus group participation.

Participants expressed a wide range of perspectives and experiences, identifying many facets of interventions and protocol needing improvement in terms of their relative advantage and adaptability for CYSHCN. Reflecting trends established in the literature, there was thorough discussion of the inaccessibility of interventions implemented to mitigate the spread of COVID-19. For example, CYSHCN had difficulty wearing masks because of breathing, sensory, or communicative challenges, faced behavioral/sensory overwhelm participating in public test or vaccine sites, were unable to receive vaccines because of physical health vulnerabilities (e.g., triggering seizures), or could not follow directions related to social distancing because of hearing or cognitive challenges.

Beyond these limitations inherent to the design of interventions, many caregivers also discussed decisions made in their implementation, and particularly in moments of transition—for instance, in the development and enforcement of rules surrounding these interventions—that created undue stress or harm for their CYSHCN. Because of the frequency of these comments, and because it is necessary to understand the transition experiences of CYSHCN in order to prepare for more equitable responses to future health crises (e.g., regardless the shape of interventions developed), we focused analyses on comments about these moments, and particularly on the relative adaptability or advantage involved. Because there was significant overlap between content labelled with these two domains (e.g., decisions that, because of their lack of adaptability, were disadvantageous to families), we do not separate the results accordingly. Instead, we present three themes developed through the process of thematic analysis: (1) “*Our kiddos didn’t have a plan when this happened*:” Lack of transition support into isolation meant loss of necessary structures and services; (2) “*He couldn’t comprehend*:” Transition communication, particularly surrounding mandates and protocol, was not handled well for CYSHCN; and (3) “*Listen, we’re still in the middle of pandemic*:” Transition timing neglected consideration of CYSHCN.

In the following sections, we describe these themes in more detail with exemplar quotes from twelve of the fourteen (86%) focus groups. To protect participant identity, all quotes are fully de-identified. Of the 77 focus group participants, sixteen (21%) are cited in eighteen quotes below, none more than twice.

### 3.1 “Our kiddos didn’t have a plan when this happened:” Lack of transition support into isolation meant loss of necessary structures and services

Children and youth with special healthcare needs experienced stay-at-home orders to be particularly disadvantageous as they lost access to a range of academic, diagnostic, healthcare, mental health, and social supports. Caregivers articulated significant social, emotional, physical, and academic setbacks that caused these decisions to have overall negative impacts, particularly because of the suddenness of the loss and the lack of transition support or adequate remote services.

“I think they dropped the ball and also the expectations for kids with special needs. They asked a lot more of them without any support.”

Remote learning options were not advantageous for many CYSHCN, as they were less effective amid difficulties focusing and lack of trained in-classroom supports, or were wholly inaccessible for those with barriers such as vision or hearing loss.

Moreover, many CYSHCN relied on in-person school for important additional experiences and services, including academic support provided through Independent Education Plans (IEPs), services offered in school settings (e.g., speech therapy), and social interactions critical for developing verbal and visual communication skills. The sudden loss of these services and exposures, without sufficient transition time, adapted protocol, or, in many cases, replacement supports, was harmful for the social, emotional, and intellectual development of CYSHCN. For example, some caregivers described how the lack of diagnostic and support services typically offered in schools created slowdowns—up to multiple years—in attaining a diagnosis, early intervention plan, or IEP for their child, resulting in regression and preventing important timely intervention. Others discussed the lack of adapted support creating regression in their youth’s enunciation and communication abilities, or loss of abilities to cope in public spaces.

“My son who was two was supposed to be an early intervention and having speech therapy and he lost it all. He now, currently as a four-year-old, you still cannot understand him. I have to translate a lot of what he says because he lost his entire speech therapy … He was so far behind because of COVID and everything. They didn’t have the testing, they didn’t have the meetings, they didn’t have anything.”

Further, because of the abruptness of the transition into remote learning, and the lack of at-home or distanced adaptations, many families lost access to these services at once, exacerbating stress and burden. One caregiver suggested that students—and particularly those with special healthcare needs—should have received an extra year of education, rather than being passed on to the next grade, given the setbacks and time lost.

“one of my sons did not get his services for the first three months … [we] had a severe reading regression. He is still not considered where he was supposed to have been.”

Beyond school, the lack of adaptation in the transition into isolation meant that CYSHCN also lost consistent or in-person access to important therapeutic services. For example, youth with physical and developmental disabilities lost access to physical and occupational therapy, halting progress or causing regression in even activities of daily living.

“My son received no vital services, which was understandable, but it set him back so far.”

Other CYSHCN, already vulnerable to stress, anxiety, and mental health challenges because of their needs, were unable to receive necessary therapy or found remote counseling ineffective.

“it really disrupted early intervention. I’m trying to Zoom a therapist to an autistic child.”

“my daughter, she was in a psychiatric facility for most of the time, and it was due to all the lack of services, the lack of school, and virtual. Our kiddos didn’t have a plan when this happened. There was no plan in place to keep them out of the hospital.”

Considering resources that provided support through these transitions, a few caregivers described the helpfulness of the caregiver-specific resource/information telephone line that was provided in Rhode Island/the U.S. and mentioned that more adapted resources like these (in other states, or a line specifically for caregivers of CYSHCN) would be helpful. Others urged advocacy and planning for future health crises that takes into account the necessity of smooth transitions and continued services for their youth.

“I definitely think advocacy if we end up in this type of a situation again, the isolation was significant. Obviously, you lose all the support. You lose your PT, you lose your speech. You lose your OT.”

### 3.2 “He couldn’t comprehend:” Transition communication, particularly surrounding mandates and protocol, was not handled well for CYSHCN

Many of the interventions and protocol implemented to stop the spread of COVID-19 were put in place as sudden wide-reaching mandates without structures for adaptation. Caregivers of CYSHCN described the trauma, stress, and cognitive incomprehensibility inherent to this lack of transition time. For example, many caregivers of youth diagnosed with autism, obsessive compulsive disorder, or otherwise exhibiting binary thinking patterns, described difficulties in comprehending the vastness of COVID-19 and related events. Without tools or time to ease into the pandemic, some CYSHCN experienced undue stress about a terrifying “green monster” suddenly significantly shaping their lives and forcing them to stay inside. Others could not comprehend the virus at all, and experienced frustration at the abundant restrictions that appeared without warning and, to their comprehension, without cause.

“His life came to a sudden halt. He knew nothing of what was going on. He couldn’t comprehend. He couldn’t understand.”

“He didn’t quite understand this invisible virus. He was… pent up and frustrated …. He didn’t understand why nobody was at the court, why things were being canceled, and why he had extremely limited hours. He took it really hard.”

Many CYSHCN also had difficulty interpreting protocol because no support was provided in communicating with them about COVID-19 or easing them into pandemic-safe behaviors. Some were unable to comprehend, and thus to follow, mandates, limiting their ability to participate in in-person services, school, or even public transportation. Other CYSHCN understood the mandates, but because of concrete thinking patterns, restricted themselves beyond necessity for fear of breaking rules. Many experienced mental and physical health difficulties as a result.

“I have a couple [of children] who have OCD, and that’s been really difficult to tell them that, ‘No, you’re outside. You can take the mask off now.’”

This lack of transition was just as, if not more, difficult for the changing and lifting of mandates as for their implementation. Throughout the years of COVID-19, rules related to masking, social distancing, and quarantining changed dramatically, at times decreasing strictness (e.g., from 14 to 10 to 5 days of quarantine) and at times increasing (e.g., mask mandates reinstated after initially being lifted) based on the severity of contagion in certain contexts and moments. Caregivers described the confusion and difficult emotions their CYSHCN experienced through these transitions. They stressed that changes were made often without warning or transition period, contingent on contexts, and with minimal explanation or adaptability.

“[My son said,] ‘Mommy, what happened with COVID?’ I don't know what to say to him because he’s saying, ‘Mommy, remember why everyone wear a mask? … Nobody in my school wear a mask. What happened with the green monster outside that keep us locked for many, many days?’”

A few caregivers also described the emotional difficulty their CYSHCN experienced as a result of the change from remote to in-person schooling, implying that there should have been more support for students through these transitions.

“The day before, it’s ‘You wear your mask, or you could die or someone around you could die.’ Then by Monday, ‘Oh wait, all the rules have changed.’ I think that’s extremely hard for someone with autism who’s practical and literal and there was no, in my opinion, real social, emotional, healthy transition for all the kids to navigate.”

Reflecting the harm of these transitions, particularly on CYSHCN, a few caregivers also expressed appreciation for instances wherein rules were adapted based on children’s needs, for example not mandating mask-wearing in classrooms when their child was having trouble.

### 3.3 “Listen, we’re still in the middle of pandemic:” Transition timing neglected consideration of CYSHCN

In addition to communication surrounding the transitions into less-severe or “post” pandemic situations, many caregivers expressed frustration that decisions to remove mandates or resources were often made without consideration of CYSHCN communities, and were not advantageous because they put their youth at disproportionate risk. The most commonly mentioned among these was the lifting of mask mandates: Many caregivers articulated a seemingly communal sacrificing or ignoring of the immunocompromised community when decisions were made to lift mask mandates.

“I'm like, ‘Listen, we're still in the middle of pandemic, still six feet back.’ … I'm not going to make everyone around wear a mask. That’s what I'm comfortable with me and my family doing, and I don't want to put that on other people, but I also feel like we still need to know that we're still in this thing.”

Many continued masking beyond mandates, sent their youth to school as the only masked person in the room, or even moved their youth to homeschooling to prevent exposure. Exacerbating the challenge of enforcing masking among youth, much less those with special healthcare needs, peer pressure and the lack of uniformity created when mandates were lifted made mask-wearing particularly difficult. Some caregivers experienced judgement when they or their CYSHCN wore masks, stigmatizing the decisions that were necessary for their CYSHCN’s safety.

“If you make it an option, then people are not going to wear them if they have the choice not to wear them. I still want my son to wear them because he was getting sick every two weeks going to school. I think they shouldn’t make it optional. It’s either everyone don’t wear them or everyone has to wear them, because then he was the only kid with the mask down in the class and then was like, ‘Oh, why do I have to wear a mask and nobody else is wearing a mask?’”

“He’s the only kid in school pretty much that wears a mask even though kids are going home with COVID and getting sick with it.”

Second most commonly, caregivers expressed frustration at the removal of publicly accessible test sites, including those catered to youth, those offering PCR tests, and those that were drive-through. While some caregivers found these sites less helpful, or even inaccessible, for their CYSHCN (e.g., overstimulation among youth with sensory sensitivities), others saw them as necessary or the only accessible option.

[in response to another participant’s plea to reopen drive-thru test sites] “I second that. Thought they were a lot more convenient. It’s a lot easier, especially if your child is disabled. Getting a wheelchair in and out of the car multiple times to do a walk-in site is not something that’s easy to do.”

One caregiver also expressed frustration at the end of state-wide tracking programs, saying that they still needed that information to make decisions in protection of their CYSHCN’s health.

Some caregivers considered the lack of flexibility in the lifting of rules a key determinant of their CYSHCN’s safety. They expressed frustration that their youth were not considered in planning, and urged such consideration, if not the development of de-implementation plans that functioned on a delayed or intermediate status to protect CYSHCN.

“In our town when things were changing, around early March, and they made the decision to make the masks optional, I felt that there should have been more input from the students in the special needs community. I actually reached out to our superintendent and the members of the school committee to say, I understand that they did have some input from the district pediatrician but it tended to focus more on the neurotypical kids. I just -sent an email to the principal at my son’s school and said, ‘Could those who are working in close contact with him, like during the therapies, please wear it?’ She said, ‘No, it’s optional now, so if they choose to, but I can’t require them.’”

Caregivers urged that, in future pandemics, the health of the special healthcare needs community be considered when planning for transitions out of the pandemic, and that resources and protection protocol be available for according to the needs of CYSHCN. The findings from this study could be used by public health response teams to proactively develop strategies to develop more advantageous and adaptive intervention transition protocol.

## 4 Discussion

It is critical to understand the experiences of CYSHCN through the pandemic in order to prepare protocols and resources that reduce physical and emotional harm among, and provide better protection and support to, these communities in future health crises (10, 12). Few studies have examined implications of the decisions implemented at the level of intervention dissemination, and especially in moments of transition (11, 13). Our study is unique in these attunements, and in that we collected primary data from CYSHCN community members on their full COVID-19 experiences. This allowed us to gather perspectives on the development, implementation, and de-implementation of interventions broadly (i.e., from stay-at-home orders, to vaccine programs, through the lifting of mask and distancing mandates), rather than limiting to one intervention or conducting a review of established data.

Through thematic analysis of focus group discussions, we have identified that CYSHCN saw decisions made in these processes as advantageous and disadvantageous, adaptable and non-adaptable, in multiple ways. We generated three themes to describe these experiences, describing that community members perceived: (1) a lack of transition support into isolation, leading to a loss of necessary structures and services; (2) mental and behavioral challenges because of poor transition communication, particularly surrounding mandates and protocol; and (3) physical and social health risks and harms because transition timing failed to consider them. These findings support previously established work in more focused contexts and extend that knowledge by identifying aspects of pandemic protocol that negatively impacted the physical, educational/cognitive, and emotional/mental health of CYSHCN and elucidating factors critical to protecting these youth in future healthcare crises. The findings can therefore be used to design protocol for future pandemic transition moments that are more advantageous for and adaptable to the CYSHCN community.

### 4.1 Physical health

Across the identified themes, caregivers expressed that decisions made surrounding COVID-19 protocol transitions created disadvantages for the physical health needs of their CYSHCN. Our findings that CYSHCN lost access to important in-person services and supports offered by schools and clinics (e.g., physical and occupational therapy) provide family-perspective reinforcement for the results of multiple previous studies (11, 13, 14). For example, Gigli et al. (13) found that CYSHCN lost access to essential specialist care, and Cohen et al. (10) highlighted widespread disruptions in developmental screenings. Similarly, Honsberger et al. noted that many state Medicaid programs struggled to adapt services (14). Furthermore, a scoping review established that nearly one-third of youth with disabilities in the U.S. “lost all rehabilitative services” (21), and a focus group study of service providers discussed the widespread loss of services necessary for families of children/adolescents with neurodevelopmental disabilities (9). Participant experiences of late-diagnosis and delayed early intervention also support similar findings in the literature (21). Further, our results support those of a few studies noting difficulties with focus, technological issues, and visual or auditory communication among CYSHCN attempting to receive remote medical care (3, 21).

Moving beyond established literature, participants in the present study described disadvantages of the early removal of masking, distancing, and related protocol necessary for health. These losses were, in some cases, repeated iteratively as the pandemic status fluctuated and intervention protocol lacked adaptability. These concerns align with Warren et al. (12), who emphasize the lack of policy adaptability for high-risk groups during pandemic transitions. While prior research noted the general sentiment that high-needs communities were overlooked in essential service decisions (10, 14), our study provides retrospective lived experiences that validate these claims. While a few studies published before de-implementation have noted the general sentiment that high needs communities felt forgotten in decisions on the relative “essential” value of certain services (9), one hypothesized the unique risks of sending CYSHCN back to school because of the hands-on care they require (8), and the lived experiences of these decisions (i.e., retrospectively) have not been discussed. The specific physical health implications of implementation and de-implementation established here support the hypotheses of previous literature discussing relative disadvantages, including physical and developmental setbacks, mental health challenges, and financial and related insecurities (9, 21). Reflecting these needs, and the recommendations of caregivers in the present study, authors have called for increased state support, adaptability in service offerings during crises, and increased clarity and accessibility in the infrastructures through which services are offered (9).

### 4.2 Cognitive and educational well-being

Caregivers described how decisions in the implementation, de-implementation, and communication of protocol were carried out in a way that was disadvantageous for the cognitive and educational health of CYSHCN. Participants' reflections on decisions regarding school closures and remote learning aligned with previous reports on how such actions were particularly detrimental to the cognitive and educational development of CYSHCN (11, 13). While such discussions are less thoroughly established in the literature, our findings do support those of a few important studies. Paralleling the inadequacy of virtual platforms for physical health care, caregivers described how CYSHCN were directly affected by the sudden removal and inadequate remote replacement of the school structure. This supports discussions of difficulties maintaining focus and lack of necessary accessibility (e.g., sign language interpreters) in remote education, preventing engagement and learning among CYSHCN (3, 9, 21). Not extensively discussed in the present population, previous studies have also noted the increased accessibility granted by remote offerings, allowing for individuals to receive care across geographic barriers, increasing caregiver involvement in care, reducing distractions typical of classrooms, and ensuring continuity of care instances wherein it is sufficient (3, 9, 21).

Caregivers in the present study acknowledged many important educational services beyond basic teaching, discussing the added burden of playing intensive physical, educational, and emotional support roles because of the loss of school-sponsored support staff, individualized education plans/supplements, and related accommodations. These findings extend similar results reporting on earlier moments (e.g., wherein data collection ended in September 2020—(3, 9, 21) by establishing that the lack of adapted educational tools extended throughout the pandemic. Beyond educational contexts themselves, caregivers also described the cognitive challenges related to pandemic communication itself, as protocol were implemented and changed in ways incomprehensible to CYSHCN. This finding has not been well-established in previous literature, though it complements arguments made by Croft and Fraser (21) on the poor operability rating of the World Health Organization’s website for adults with intellectual and developmental difficulties. These challenges affirm the necessity of considering communities with cognitive difficulties when developing virus prevention protocol, when adapting educational offerings according to those protocol, and when developing public messaging about them.

### 4.3 Emotional and mental health

Intertwined with and compounding physical and cognitive difficulties, caregivers expressed that the transition periods of COVID-19 protocol were handled in such a way that they harmed the emotional and mental health of CYSHCN. The disruption of socialization and communication structures created emotional distress, was consistent with prior studies documenting increased anxiety, depression, and behavioral challenges (10, 12, 13). In protocol implementation and changes, the unique socialization and communication needs of this community were neglected in isolation mandates and remote options without adaptability. Similar difficulties are most thoroughly described in the global scoping review, which discusses the negative implications of the loss of structure and “disruption of everyday life” that many youth experienced (21). Our findings affirm the specific effects of isolation experienced across the community that Croft and Fraser (21) identify, such as anxiety, depression, difficulty sleeping, and behavioral issues. Our findings also extend the work of Warren et al. (12), who emphasize that CYSHCN were frequently overlooked in public health messaging and policy decisions. They also support findings discussing the difficulties of virtual forms of mental health care for CYSHCN, reiterating the aforementioned challenges associated with communication, attention, and accessibility (3, 9, 21).

Additionally, caregivers in this study identified inflexible mass-communication, unexplained fluctuation of protocol, and mandates that limited possibilities for adaptation or safe enforcement as creating specific threats to their CYSHCN’s emotional health. Our findings support early arguments by Fontenelle and Miguel (34) that youth with obsessive compulsive disorder and related diagnoses experienced significant self-restriction and stress because of public messaging about cleaning habits and protocol. Experiences of stress and confusion in the wake of fluctuating expectations also support previous discussions of the importance of consistency and routines among youth with diagnoses like autism spectrum disorder (21, 30). Because of the later moment of data collection, we add to established literature recognition of the social and emotional implications of protocol removed too-early and without-adaptation, in that youth whose physical vulnerabilities required that they continue wearing masks or practicing distancing after mandates were lifted faced not only established threats of contagion from unmasked peers, but also peer pressure and social challenges, contributing to poor emotional well-being or to unsafe decisions (i.e., to unmask). Each of these implications, while specific to diagnoses that impact youth’s experiences of rule communication, changing, and universal applicability, urge adaptation and flexibility in order to protect mental and emotional health.

### 4.4 Recommendations

We present these experiences of CYSHCN not as a complete summary of needs, but that we might signal to the implications of a larger silencing at work in the past development and implementation of health crisis interventions. As other studies (7, 10–12, 31) have illustrated, no description of pandemic experiences is unanimous. In this sample, perspectives varied widely, and even created contradictions, across personal situations, forms of disability/high needs, political orientations, and community environments. Regardless of this inconsistency, and because of the breadth of the inaccessibilities apparent across the sample, our findings have supported and extended the literature in establishing that caregivers perceived the physical, cognitive, and emotional health of their CYSHCN to have been neglected in the development, implementation, and de-implementation of pandemic prevention protocol (1, 8, 21). We therefore support other authors in urging the recognition of the unique needs of the CYSHCN community (1, 21) in designing and implementing multilayered, adaptable, and flexible pandemic prevention protocol (8, 21).

Narrowing in on the transition moments of the COVID-19 pandemic, and particularly on the relative advantage and adaptability of implementation processes, allows us to identify specific opportunities for improvement. Implementation scientists provide general guidance on addressing these intervention characteristics, which we will expound upon specifically below. First, scholars have described the importance of developing interventions that can be adapted both to community member needs and to the local culture or infrastructure (26, 27). This process involves intentionally evaluating the characteristics of an intervention that are necessary for its efficacy and those that can be changed (32). Accordingly, this process must be carried out specifically, considering each intervention and its mechanism individually as they relate to the local community. Second, authors have noted that the perceived relative advantage of an intervention is a “sine qua non” for effective implementation, urging that the benefits of a tool or decision should be clear and unambiguous (27). Perceived relative advantage exists beyond evidence-bases for efficacy and efficiency, and often is determined by factors such as framing, perceived consequences, and local meaning-making or prioritization (27). Like determining adaptability, the process of increasing perceived relative advantage necessitates intentional engagement, particularly with high-needs community members, to understand local community needs, conceptualizations of impact, and readiness for change (27).

Noting these needs, further research, particularly that which empowers people in CYSHCN communities as study leads and key informants, should be conducted to clarify and further expound on each of these. A key tenet of inclusive work is the empowerment of disabled community members, not only as research participants, but as leaders in the development of studies and knowledge (33). These methods are critical to implement across policy-development and, our findings suggest, are particularly important in caring for the CYSHCN community. Based on our findings, we propose three key research and policy protocol in preparation for future pandemics. These can be usedto guide the development of proactive approaches to meeting the needs of CYSHCN communities in transition moments while ensuring effective public health intervention deployment:

1. Developing messaging around policy changes due to public health emergencies. Empowering the perspectives and experiences of CYSHCN community members will aid in developing communication during public health emergencies that identifies and frames the relative advantages of a decision according to the needs and preferences of the local community. This will also support development of messaging that is comprehensible across cognitive abilities (21), which minimizes the emotional burden created by mandates (e.g., inciting stress or excessive-restriction), and which provides families with the information needed to protect their CYSHCN (e.g., addressing the specific needs of these communities at regular press conferences).
2. Creating infrastructural support for flexibility and adaptation. While adaptations like virtual services and clear masks during the COVID-19 pandemic provided significant benefits, they were not fully effective, and leaders need to adapt the tools and the policies enforcing them according to community needs. For example, participants and previous researchers have called for intentional consideration of the specific kinds of appointments and patient populations for which virtual platforms are suitable (e.g., best for those without physical and tactical interaction), and for exception or adaptation [“a blended model” (3)] for the cases in which remote care is not functional (3, 21). Involvement of CYSHCN community members will allow researchers and policy-developers to identify the specific instances wherein adaptations are in/sufficient, and to draft flexible protocol, granting clear allowances and expanding adaptations to ensure that essential needs are met.
3. Adding buffers, supports, and delays during de-implementation. Transitions into and out of phases of public health crises should be just that: transitions; and they should be adaptable to varying definitions of safety in order to be advantageous to varying communities. In addition to flexibility within mandates themselves, mandates should be implemented gradually, with supports and structures for iterative feedback—i.e., opportunities for CYSHCN community members to identify factors forgotten in policy-development, and for those factors to be addressed broadly and quickly. This applies also to de-implementation: it is essential that the health and well-being of CYSHCN communities be taken seriously in decisions to remove safety measures, and that removals be gradual to ensure extended protection.

### 4.5 Limitations

In this study, we aimed to illustrate the breadth of experiences through COVID-19 across a wide range of experiences; this breadth presents limitations in that we are unable to present trends and recommendations specific to diagnosis, child’s age, setting (e.g., school or healthcare), or socioeconomic and/or cultural context. Future research might examine perspectives specific to these factors in order to strengthen findings and inclusive policy development. Similarly, in seeking a full-scope retrospective perspective of the COVID-19, focus groups asked that participants rely on memory for specific details of early pandemic experiences, This risks inaccuracies in data, which we sought to mitigate through the large number of focus groups and participants included in the study, with trends and conclusions only drawn from repeated comments. Additionally, in recruiting participants in partnership with a local non-profit organization, we may have neglected the perspectives of families without access to such support, thus experiencing even more significant resource and accessibility needs than expressed here. Further, the presence of the non-profit staff member during the focus groups might have prevented caregivers from sharing freely, even as the staff member’s identity as a caregiver of a CYSHCN might have mitigated this effect; the breadth of responses suggests caregivers were not wholly limited.

## 5 Conclusion

This study presents analysis of fourteen focus groups conducted among 77 caregivers of CYSHCN. Analyzing retrospective accounts of experiences during COVID-19, we posit that it is critical to focus on moments of transition in pandemic interventions, and we describe trends in caregiver experiences of relative advantage and adaptability through three themes: (1) “*Our kiddos didn’t have a plan when this happened*:” Lack of transition support into isolation meant loss of necessary structures and services; (2) “*He couldn’t comprehend*:” Transition communication, particularly surrounding mandates and protocol, was not handled well for CYSHCN; and (3) “*Listen, we’re still in the middle of pandemic*:” Transition timing neglected consideration of CYSHCN. In the development of pandemic protocol and implementation of intervention decisions, the needs and vulnerabilities of CYSHCN were neglected, creating negative implications for their physical, educational/cognitive, and social/emotional health. We propose that inclusive and adaptive models of punlic health research and policy development are critical to creating transition plans that are congruent with community needs and challenges.

## Data Availability

The data sources supporting the conclusions of the manuscript were the transcripts of fourteen focus groups conducted with 77 parents of CYSHCN. For participant protection, particularly given the vulnerability of this population and the information they shared with us (e.g., rare diagnoses that might be identifying in a small community) focus group transcripts are not publicly accessible. Anonymized transcripts (or excerpts) generated and analyzed for this study can be provided upon request to the corresponding author. Requests to access the datasets should be directed to Neil Sarkar, neil_sarkar@brown.edu.
